# Bow Hunter's syndrome with clicking sounds: A rare etiology of transient loss of consciousness with tonic–clonic seizure

**DOI:** 10.3389/fneur.2022.1088842

**Published:** 2023-01-12

**Authors:** Lijuan Wang, Yanan Dong, Hongxiu Chen, Jing Bai, Mingqin Zhu, Yingqi Xing

**Affiliations:** ^1^Department of Neurology, The First Hospital of Jilin University, Changchun, China; ^2^Department of Vascular Ultrasonography, Xuanwu Hospital Capital Medical University, Beijing, China; ^3^Beijing Diagnostic Center of Vascular Ultrasound, Beijing, China; ^4^Center of Vascular Ultrasonography, Beijing Institute of Brain Disorders, Collaborative Innovation Center for Brain Disorders, Capital Medical University, Beijing, China

**Keywords:** Bow Hunter's syndrome, vertebrobasilar insufficiency, carotid artery ultrasound, epileptic seizure, etiology

## Abstract

We present the case of a young male patient experiencing a transient loss of consciousness and manifesting a seizure when he tilted his head backward. Transcranial Doppler ultrasound (TCD) and carotid artery ultrasound (CAU) examination were normal when the patient's neck was in the neutral position. However, the CAU revealed vertebral artery (VA) transient occlusion during neck rotation or backward movement. Electroencephalogram (EEG) monitoring was performed with multiple neck rotation–induced tests. The patient developed dizziness, which was the same as the prodromal symptoms of the first seizure, and the EEG showed a large number of spinal slow waves and sharp slow waves in the frontal-to-frontal midline area, with an occasional generalization trend. CT angiography revealed occipitalization of the atlas and the lack of contrast agent filling in the local area of the VA when the patient's head was turned contralaterally. Thus, the patient was diagnosed with Bow Hunter's syndrome (BHS) and treated conservatively with neck immobilization. No recurrence occurred at 3 and 6 months of follow-up. Therefore, this case alerts neurologists to suspect BHS on observing seizure manifestations during neck rotation, and CAU may be a recommended dynamic screening method for BHS. This report is accompanied by a discussion of the phenomenon and diagnosis in the context of the existing literature.

## Introduction

Bow Hunter's syndrome (BHS), also known as rotation vertebral artery (VA) occlusion syndrome, is a rare disorder. It is characterized by vertebrobasilar insufficiency (VBI) caused by transient rotational VA compression during head movement. This syndrome involving vertigo and nystagmus caused by the head movement was first described by DeKleyn and Versteegh in 1933 (1) and was named by Sorensen as Bow Hunter's syndrome, to describe a man who experienced brain stem infarction during archery practice in 1978 (2).

The primary symptoms of BHS are syncope, pre-syncope, vertigo, drop attacks, intermittent dizziness, headache and paresthesias, double vision, and impaired vision (3, 4), which usually resolve immediately when the head returns to a neutral position. Furthermore, bone spurs or osteophytes are the most common etiology of primary BHS (5). Another type is acquired BHS, which is caused by complications of cervical spondylosis, surgery (6, 7), and neck or head injury (8, 9). Previous studies have reported that BHS is more common in the left VA. The higher involvement of the left VA may be due to the left VA dominance, accounting for 50% of cases, whereas the right VA dominance occurs in only 25% of cases (5, 10, 11). Hence, the left VA is more often occluded than the right VA in patients with BHS. However, the occlusion of bilateral VAs has been shown to occur rarely in BHS (12–14).

In this study, we report on a patient with BHS and bilateral VA insufficiency who presented with transient loss of consciousness and seizures. This study provides rare clinical information and an etiology for BHS. Furthermore, we summarize the characteristics, symptoms, and evaluation of patients with this rare syndrome.

## Case report

A 29-year-old man was experiencing transient loss of consciousness and generalized tonic–clonic seizure associated with rotation of the head. Then, he recovered consciousness with a slow response, ~3 min later. He experienced seizures once again for 4 min during neck rotation when he visited the hospital. The patient had a long history of complaints of headaches and dizziness since childhood. He had no history of hypertension, diabetes, or heart disease. Furthermore, he had no family history of similar complaints, vascular or connective tissue disease, stroke-like episodes, seizures, or early-onset dementia. However, he experienced clicking sounds at the back of his neck when he tilted his head backward.

**Figure 1:**
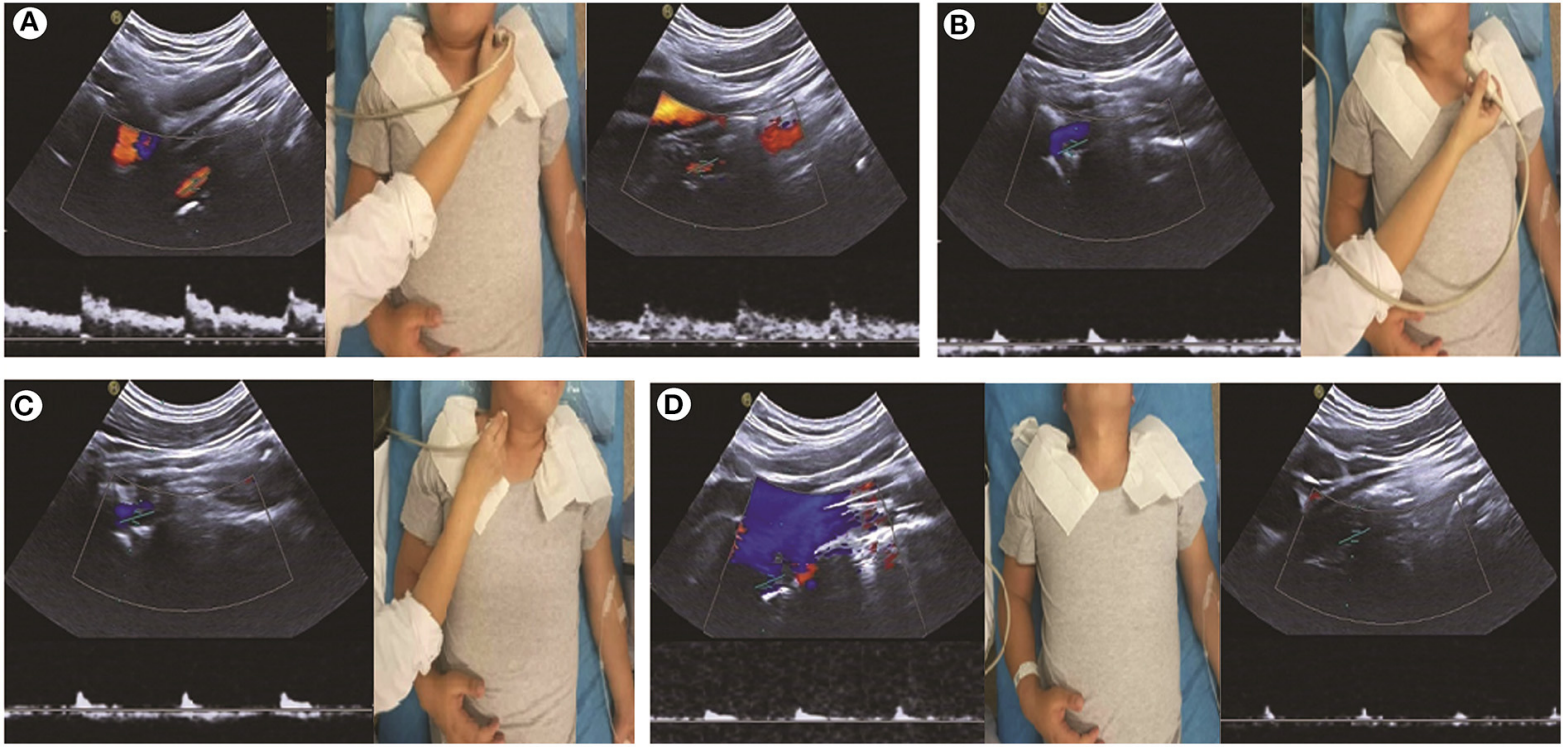
Carotid artery ultrasound shows abnormalities during neck rotation. **(A)** The velocity of blood flow and spectrum of the bilateral vertebral artery (VA) is normal with the patient's neck in the neutral position. **(B, C)** Low bloodstream and high resistance during the head rotation to the contralateral side, indicating that the distal part of the VA is occluded. **(D)** Bilateral VA is occluded when the head is tilted backward.

**Figure 2:**
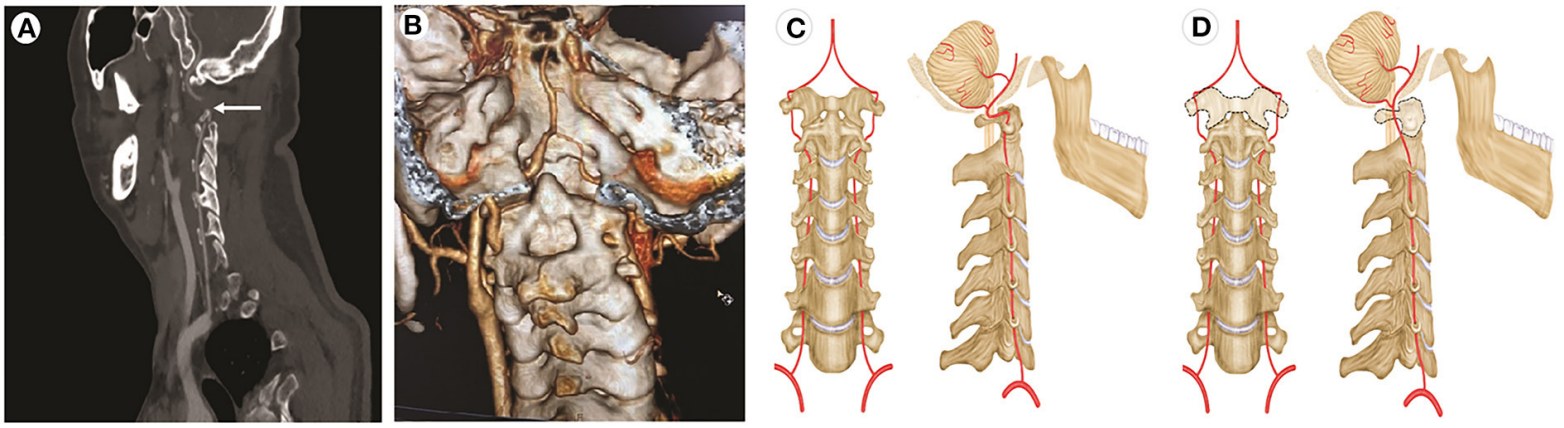
CT angiography and illustrations of normal VA and potential cause of BHS involving occipitalization of the atlas. **(A)** A contrast agent is not filling the local area of the right vertebral artery (arrow) with the patient's head turned left. **(B)** CT angiography reveals occipitalization of the atlas (C1) and congenital agenesis of the posterior elements of the atlas (C1). **(C)** Normal movement of the VA. **(D)** The dotted line shows the dysplastic atlas.

Physical examination, CT, and MRI did not reveal any abnormalities. The cerebrospinal opening pressure on the lumbar puncture and cerebrospinal fluid routine examination were normal. The electroencephalogram showed a large number of spinal slow waves and sharp slow waves in the frontal-to-frontal midline area, with an occasional generalization trend. Those abnormalities were considered to be related to previous seizures. The transcranial Doppler ultrasound (TCD) and carotid artery ultrasound (CAU) showed a normal blood flow velocity and resistance index (RI) in the bilateral VA when the patient's neck was in the neutral position. However, the CAU revealed abnormalities during neck rotation or backward movement. The peak systolic velocity (PSV) in the VA decreased, end-diastolic velocity (EDV) disappeared, and RI increased when the patient's head was turned to the contralateral side. The EDV disappeared and RI increased, which indicated that the distal part of the measurement point in the artery was occluded. The PSV in the bilateral VA decreased, EDV disappeared, and RI increased when the head was tilted backward (Figure 1). CT angiography (CTA) showed the right VA was thinner than the left VA, and no clear contrast agent filling was observed locally in the V2 segment of the right VA in a specific left-turn cervical position. The right posterior communicating artery was dilated. Multi-slice spiral CT image reconstruction of the cervical spine revealed the disappearance of the atlantooccipital space, occipitalization of the atlas, and cervical 2–3 vertebral body fusion (Figures 2A, B). The atlantooccipital joint is the major site responsible for neck rotation. We presume that the occipitalization of the atlas caused instability, resulting in the patient hearing clicking sounds. This patient had occipitalization of the atlas, an instability attributed to congenital cervical abnormalities, which increases the likelihood of arterial compression during head rotation (Figures 2C, D). Finally, the patient was diagnosed with Bow Hunter's syndrome (BHS) and was treated conservatively with neck immobilization and aspirin. No recurrence occurred at 3 and 6 months of follow-up.

## Discussion

Here, we presented a rare case of BHS in which the patient experienced clicking sounds and bilateral VA compression. BHS could have been caused by the occipitalization of the atlas, which has not been reported previously.

### Etiology and pathogenesis

Vertebrobasilar insufficiency in BHS is due to compression of the VA owing to various etiologies, such as bone spurs, osteophytes (5), herniated disks, osteophytic lesions, uncovertebral instability, or osseofibrous bands (15, 16). The VAs ascend across six foramina transversarium of the cervical vertebrae, pass through the groove on the surface of the arch of the atlas and then through the foramen magnum, and enter the skull (17). Anatomically, the compression of the VA could occur at all vertebral levels; however, the high cervical spine (C1-2) was most commonly observed to be involved (5, 10). Nevertheless, a literature review showed that arteries were mostly occluded between C3 and C7 (58%), followed by C1–C2 (36%), and then, proximal to C7 and distal to C1 (6%), which is the most unusual position (3). Bone spurs or osteophytes are the most common etiologies of primary BHS (5). Of note, in our patient, CTA revealed occipitalization of the atlas, and this case is very rare, as no such cases exist in the literature.

The atlantooccipital joint is the major site responsible for neck rotation. Furthermore, the VA is relatively fixed at the exit of the C1 transverse process and the entry into the atlantooccipital ligament; these anatomical relationships make the VA prone to occlusion or injury at this site. Our patient had occipitalization of the atlas, an instability attributed to congenital cervical abnormalities, which may increase the likelihood of artery compression during head rotation (18). To the best of our knowledge, occipitalization of the atlas in patients diagnosed with BHS is extremely rare.

In addition to vertebrobasilar insufficiency, which is a major symptom in patients with BHS, our patient had other special concomitant symptoms. Our patient could hear clicking sounds occurring at the back of his neck when he tilted his head backward, which no previous study has reported in other patients with BHS. We presume that the occipitalization of the atlas caused instability, resulting in the patient hearing clicking sounds. Additionally, this patient experienced seizures and a loss of consciousness. To the best of our knowledge, seizures are a very rare symptom in patients with BHS. The seizures were probably secondary to arterial insufficiency. Seizures occur in patients with cortical infarcts in the anterior circulation. Thus, seizures associated with posterior circulation insufficiency are considered rare (19). The vertebrobasilar insufficiency was due to the compression of the VA, while the dilated posterior communicating artery was speculated to steal the blood flow from the internal carotid artery to compensate for the posterior circulation. This compensation would reduce the blood flow of the anterior circulation and result in cerebral metabolic and perfusion disorders. The potential mechanisms of epileptogenesis in cerebrovascular disease included potential interrelationships between the disorder of cerebral perfusion and metabolism, disruption of neurovascular unit integrity, dysfunction of the blood–brain barrier, and inflammation (20). A few previous studies have reported that patients with posterior circulation insufficiency experience seizures and epilepsy (21, 22).

### Examination and diagnosis

Bow Hunter's syndrome can be diagnosed using different techniques. Digital subtraction angiography (DSA), the gold standard for diagnosing BHS, can reveal a patent artery when the head is in the neutral position and a stenotic artery when the head is in the rotated position (23). However, this invasive and expensive evaluation is not suitable for the preliminary screening of BHS. Concerning non-invasive techniques, CTA can detect abnormal bony structures and stenosis of arteries. Thus, the topographical relationships between the vascular anatomy and the surrounding structures can be precisely observed on CTA in the same image (24). However, dynamic compression of the VA cannot be reproduced during CTA. In addition, during the scanning process, there may be potential risks of causing severe symptoms, even posterior circulation ischemia, when the patients rotate their heads to the symptomatic side (10). Neurosonography, including CAU and TCD, has been increasingly utilized as a non-invasive and inexpensive bedside techniques for evaluating hemodynamics. However, TCD has some disadvantages. The vascular lumen cannot be observed visually by TCD; hence, the vessels cannot be visualized directly using this technique, and the Doppler spectrum of the arteries can only be viewed by placing the probe on the common detection positions. Thus, to some extent, this may be a “blind detection” technique, and the operator will require experience and time to search for the Doppler spectrum of the arteries. In addition, owing to the patient rotating their head, the location of the probe can change, causing the signal to disappear. Therefore, a continuous change in the spectrum will be difficult to observe. Furthermore, it is difficult to determine whether the signal disappeared because of an interruption in the blood flow caused by the compression of arteries or because of a probe shift. Thus, operators will need to spend time to search for the Doppler spectrum to clarify this. Moreover, a brain stem infarction could occur, and patients could also experience fainting. Conducting a CAU can, therefore, provide more accurate results because it can display the vascular lumen intuitively.

Therefore, in this study, we used CAU, which revealed a normal RI and blood flow velocity of the VA when the patient's head was in the neutral position, and the diastolic blood flow disappeared and RI increased when the patient's head was tilted to one side or backward. Indeed, it is easier for the operator to acquire a continuous change in the spectrum because of its visual display. Furthermore, the operator could immediately detect a signal disappearance caused by the blood flow interruption or a probe shift. Hence, more reproducible hemodynamic changes and a feasible and easier detection can be obtained by using CAU in patients suspected to have BHS (25).

We present a case of BHS and bilateral VA insufficiency identified through screening using CAU, which has not previously received enough attention as a useful technique by neurologists. CAU could be a recommended method to screen for BHS. Furthermore, owing to its non-invasiveness and repeatability, this technique can also be widely used to evaluate the therapeutic effects and follow-up.

### Treatment and management

Bow Hunter's syndrome remains a rare clinical condition with no standard treatment options. Therapy includes the conservative approach, such as neck immobilization, using a cervical collar or neck brace and surgery and endovascular intervention (23). Moreover, antiplatelet or anticoagulation therapy is recommended for secondary stroke prevention in patients with BHS. Additionally, the patient should be instructed to avoid rotating the neck and head in the symptomatic direction (10). Choi et al. analyzed 21 patients with rotational VA occlusion and suggested that conservative treatments might be safe and a first-line therapy (26). Although surgical management remains controversial, another study of 153 patients with BHS reported that surgery resulted in significantly favorable outcomes (5). Endovascular treatment for BHS includes endovascular stenting of VAs or coil embolization of a symptomatic, non-dominant VA (27, 28). In this case, considering the surgical risks, the patient temporarily refused surgery and received conservative treatment. Thus, the management of BHS has not been standardized, and large randomized clinical trials are needed to evaluate the effectiveness of therapy. It was suggested that BHS treatment should be administered based on the mechanism of VA compression and symptoms and should thus vary among such patients.

## Conclusion

A neurologist should suspect BHS on observations of vertebrobasilar insufficiency during neck rotation. CAU is a recommended method to screen for BHS.

## Author contributions

YX and LW conceived and designed the manuscript. YD performed the carotid artery ultrasound. LW, HC, JB, and MZ collected the patient information. LW and HC were the major contributors to writing the manuscript. YX revised the manuscript. All authors have read and approved the final manuscript.
